# Familial ovarian screening – effective or ineffective?

**DOI:** 10.1038/sj.bjc.6603407

**Published:** 2006-10-17

**Authors:** I J Jacobs, J Mackay, U Menon, S J Skates, A N Rosenthal, L Fraser

**Affiliations:** 1 Department of Gynaecological Oncology, Institute for Women's Health, University College London, London, UK; 2 North East Thames Clinical Genetic Service, Great Ormond Street Hospital and the Institute of Child Health, University College London, London, UK; 3 Department of Medicine, Harvard Medical School, London, USA

**Sir**,

We would like to comment on the paper (Oei *et al*, 2006) which we feel is misleading as it over-interprets data from a very small study. The authors describe the discovery of two cases of ovarian cancer during 1029 women years of screening. One case was a stage 3c cancer, however, this was detected at the time of the woman's first screen. This was therefore a prevalent case, the stage of which could not be influenced by screening, and so no conclusions about the efficacy of screening can be drawn from it. The second case was a stage 2b cancer found at prophylactic bilateral salpingo-oophorectomy 4 months after an apparently normal transvaginal scan and CA125 result. This case would be consistent with the suggestion from a recent meta-analysis (Hogg and Friedlander, 2004), that annual screening may not have adequate sensitivity for the detection of early stage disease. The authors of the meta-analysis noted the lack of reliable data in the high-risk population to address this issue and advocated prospective trials in women at increased genetic risk. Unfortunately, the study by Oei *et al* is not adequately powered for this purpose.

Despite Oei *et al*'s assertion that high risk population surveillance is ‘inefficient’, there remains a need for screening among younger women in this group, who understandably wish to delay prophylactic surgery in order to have children or to avoid premature menopause. This is evidenced by the fact that 60% of the women in the UK Familial Ovarian Cancer Screening Study (UKFOCSS) are aged under 50 years (Rosenthal AN, personal communication). We have recently obtained funding from Cancer Research UK and the Eve Appeal to screen a high-risk population of 3000 women for 5 years using 4 monthly rather than annual CA125 testing (phase 2 of UKFOCSS). The CA125 results will be analysed using the risk of ovarian cancer algorithm (ROCA) (Skates *et al*, 2001), which has been successfully piloted in the postmenopausal general population (Menon *et al*, 2005). This algorithm has been adapted for use in the premenopausal population and it is hoped that it will improve sensitivity and specificity for early stage disease detection. UKFOCSS is on target to complete recruitment by the end of 2006. There is only one other large-scale study of high-risk population screening recruiting worldwide. This is the US Cancer Genetics Network (CGN) study, which is also using 4-monthly CA125 testing analysed by the ROCA. The intention is to share data between the two studies, even though each study is sufficiently powered to assess the performance of screening in terms of sensitivity, specificity and positive predictive value.

Until UKFOCSS and the CGN study report in 2012, there is unlikely to be sufficiently reliable evidence to conclude that familial ovarian cancer screening is effective or ineffective. We therefore recommend that high-risk women in the US and UK unwilling to undergo prophylactic bilateral salpingo-oophorectomy take part in these studies if they wish to undergo screening.
